# The Importance of microRNAs in RAS Oncogenic Activation in Human Cancer

**DOI:** 10.3389/fonc.2019.00988

**Published:** 2019-09-27

**Authors:** Roberta Roncarati, Laura Lupini, Ram C. Shankaraiah, Massimo Negrini

**Affiliations:** ^1^Department of Morphology, Surgery and Experimental Medicine, University of Ferrara, Ferrara, Italy; ^2^CNR, Institute of Genetics and Biomedical Research, National Research Council of Italy, Milan, Italy

**Keywords:** microRNA, RAS, cancer, MAPK, target therapies

## Abstract

microRNAs (miRNAs) regulate gene expression by modulating the translation of protein-coding RNAs. Their aberrant expression is involved in various human diseases, including cancer. Here, we summarize the experimental pieces of evidence that proved how dysregulated miRNA expression can lead to RAS (HRAS, KRAS, or NRAS) activation irrespective of their oncogenic mutations. These findings revealed relevant pathogenic mechanisms as well as mechanisms of resistance to target therapies. Based on this knowledge, potential approaches for the control of RAS oncogenic activation can be envisioned.

## Introduction

microRNAs (miRNAs) are small (19–24 nucleotides) non-coding RNAs discovered in 1993 in studies related to embryonic development of C. *elegans* (1, 2). Their importance significantly increased following the discovery of their existence in all eukaryotic organisms (3). Currently, 2,654 mature miRNAs, originating from 1917 precursors, are described in humans (http://www.mirbase.org/) (4, 5). Their main function is to negatively regulate gene expression at the post-transcriptional level through the interaction of their “seed” portion by sequence homology typically with the 3′ non-coding regions of messenger RNAs (mRNAs). Through this interaction, miRNAs limit translation, or promote degradation of target mRNAs (6, 7).

The modulation of target mRNAs by miRNAs is complex, considering that each mRNA is generally targeted by multiple miRNAs, and the strength of this interaction is variable (8). Classically, it has been thought that each miRNA can interact with hundreds of target mRNAs. However, recent reports have highlighted RNA transcripts inducing degradation of respective interacting miRNAs through a mechanism known as “target-directed miRNA degradation” (TDMD) (9, 10). Added to the complexity of these direct interactions is the fact that some long non-coding RNA (lncRNA) could function as “sponges,” that act as a buffer and prevent the action of miRNAs on target protein-coding mRNAs (11, 12). Lastly, it is also important to consider that cell co-localization of each miRNA with the target mRNAs is necessary and depends on the eventual tissue-specific expression of each of the interacting RNAs.

Thus, miRNAs, taken together, represent an essential phase in the regulation of gene expression by modulating the translation of the entire transcriptome (13, 14). Given their biological importance, their deregulation plays a significant role in pathogenic mechanisms, including the neoplastic transformation (15, 16). The first evidence associating miRNAs with human malignant diseases was the discovery of miR-15 and miR-16 in the minimal region of deletion at chromosome 13q14 in chronic lymphatic leukemia (17). Since this seminal study, a myriad of other studies has confirmed the role of miRNAs in tumorigenesis and other human diseases as well.

## miRNAs as Direct Regulators of RAS

The first functional evidence to establish a molecular link between the deregulation of miRNAs with an explicit oncogenic pathway was published in 2005 when Slack and collaborators reported the importance of the downregulation of members of the *let-7* miRNA family with the activation of oncogenes of the RAS family (18). The study demonstrated that the 3′ UTRs of KRAS, NRAS and HRAS mRNAs comprised multiple complementary *let-7a* binding sites. The enforced expression of let-7 could indeed reduce RAS protein levels (18). Conversely, let-7 downregulation could lead to the loss of its post-transcriptional control, causing RAS over-expression and activation. This study was decisive in proving that aberrant expression of miRNAs could play an important role in tumor initiation and progression, and paved the way for studies that extended miRNA involvement to all phases of neoplastic initiation and progression (19).

The involvement of RAS (KRAS, NRAS, HRAS) in human tumors is mainly associated with the presence of activating mutations at codons 12, 13 and 61, able to activate various molecular pathways, which play a key role in a large number of tumor traits, spanning from cell proliferation, cell survival, cytoskeleton organization, motility, and more (20). The demonstration of the role of miRNAs in the abnormal regulation of RAS thus represented another important mechanism involved in key steps of tumorigenesis.

Since then, quite a few other reports have demonstrated the modulation of RAS by miRNAs. In many cases, the interaction was only predicted by computer algorithms, but several studies have experimentally validated these interactions. Table 1 lists the microRNAs for which the ability to modulate the expression of KRAS, NRAS, or HRAS has been experimentally confirmed.

**Table 1:** Human microRNAs targeting RAS family members.

**miRNA**	**HRAS**	**KRAS**	**NRAS**
hsa-let-7a-5p	1	1	1
hsa-let-7b-5p	1	1	1
hsa-let-7c-5p		1	1
hsa-let-7g-5p		1	
hsa-miR-1-3p		1	
hsa-miR-16-5p		1	
hsa-miR-18a-3p		1	
hsa-miR-20a-5p			1
hsa-miR-26a-5p			1
hsa-miR-27a-3p		1	1
hsa-miR-96-5p		1	
hsa-miR-98-3p			1
hsa-miR-98-5p			1
hsa-miR-124-3p			
hsa-miR-126-3p		1	
hsa-miR-134-5p		1	
hsa-miR-139-5p	1		
hsa-miR-143-3p	1	1	
hsa-miR-145-5p			1
hsa-miR-148b-3p			1
hsa-miR-152-3p		1	
hsa-miR-155-5p		1	
hsa-miR-181a-5p	1	1	1
hsa-miR-181c-5p		1	
hsa-miR-181d-5p	1		
hsa-miR-193a-3p		1	
hsa-miR-193b-3p		1	
hsa-miR-199a-5p		1	
hsa-miR-200c-3p		1	
hsa-miR-206		1	
hsa-miR-214-3p			1
hsa-miR-216b-5p		1	
hsa-miR-217		1	
hsa-miR-224-5p		1	
hsa-miR-340-5p		1	
hsa-miR-365a-3p		1	
hsa-miR-384		1	
hsa-miR-433-3p		1	
hsa-miR-452-5p		1	
hsa-miR-487b-3p		1	
hsa-miR-543		1	1
hsa-miR-613		1	
hsa-miR-622		1	
hsa-miR-663a	1		
hsa-miR-4689		1	

*Data from miRTarBase (http://mirtarbase.mbc.nctu.edu.tw)*.

As mentioned, let-7 was the first, and probably the most important miRNA implicated in the regulation of genes of the RAS family (18). In the human genome, 12 loci are known to encode for members of the let-7 family: let-7a-1, -2, -3; let-7b; let-7c; let-7d; let-7e; let-7f-1, -2; let-7g; let-7i; miR-98. While it is described that members of the let-7 family are up-regulated in the course of cell differentiation, numerous studies have reported the reduction of let-7 expression in different tumor types (21, 22). Already in 2004, Takamizawa et al. demonstrated the downregulation of let-7 in non-small cell lung carcinoma (NSCLC) (23, 24) and documented its prognostic significance. Furthermore, in line with these observations, they proved that enforced expression of let-7 miRNA could inhibit *in vitro* cell growth of the lung adenocarcinoma A549 cells (23, 25–27). These studies were further confirmed in murine *in vivo* models of NSCLC (28, 29) and revealed that let-7 mimics could represent potential therapeutic molecules.

Given the proven interaction of let-7 with members of the RAS family, it is plausible that the observed effects were due to the modulation of RAS. However, let-7 can also regulate additional important oncogenes such as c-MYC, high-mobility group A (HMGA), Janus protein tyrosine kinase (JAK), signal transducer and activator of transcription 3 (STAT3) (30). Its action as a tumor suppressor gene is therefore achieved through the ability to interact with multiple oncogenes and inhibit the activation of their molecular pathways (18, 28).

Essentially all types of human cancer present a general down-regulation of let-7 (21). Among others, the modulation of RAS by let-7 was demonstrated in colorectal cancer (CRC) where let-7 is strongly down-regulated in tumor tissues compared to adjacent healthy tissues. Similar to the study on NSCLC cells, let-7 was also shown to act as a growth suppressor in human CRC cells (31).

Confirming the importance of RAS regulation by let-7, the discovery of the LCS6 polymorphism (Let-7 Complementary Sites 6, rs61764370) in the KRAS 3′ UTR region further demonstrated let-7 expression altering interaction. This polymorphism has been associated with a greater risk of developing tumors and worse prognosis in lung, oral, and colorectal cancer (32–34).

An understanding of a mechanism leading to let-7 down-regulation in cancer came from studies on LIN28 in mammals. Lin28 and Lin28b act as RNA binding proteins that are able to associate with the terminal loop of the precursors of let-7 family miRNAs and block their processing into mature miRNAs (35–38). Since LIN28 is over-expressed in human cancer, this mechanism causes let-7 down-regulation, which establishes a connection with RAS and other cancer-associated signalings.

Let-7 is not the only miRNA involved in the regulation of RAS (HRAS, KRAS, or NRAS) (39). Among the miRNAs involved in the regulation of members of the RAS family, miR-143 and miR-145, co-expressed in the same primary transcript, can target both KRAS and NRAS, and have been found to be down-regulated in numerous human tumors (40–42). Already in 2003 Michael et al. documented a significant reduction of miR-145 in CRC compared to normal mucosa (43) and in 2014, Pagliuca et al. confirmed that the miR-143/miR-145 cluster, highly expressed in normal colon, was significantly decreased in CRC (44). Their reduced expression has been correlated with p53 mutations capable of reducing the maturation process of these miRNAs (45).

Very similar to let-7, members of the miR-181 family were shown to target all the RAS family members (HRAS, KRAS, and NRAS). They were found downregulated in different types of cancer, such as oral squamous cell carcinoma (46, 47), gastric cancer (48), and gliomas (49). These findings suggest that miR-181 down-regulation is one of the mechanisms leading to oncogenic RAS activation in these tumors.

It is notable that in spite of KRAS activation by gene mutation in 90% of the cases in pancreatic cancer, various miRNAs capable of directly targeting KRAS are simultaneously downregulated. Specifically, miR-96, miR-126, and miR-217 (50–53). Since the reduced expression of these miRNAs correlates with higher KRAS expression, these alterations likely represent a mechanism for strengthening the already activated RAS signaling.

Another noteworthy miRNA capable of targeting KRAS is miR-134. It was found downregulated in glioblastoma and renal cell carcinoma (54, 55). miR-134 downregulation correlated with the activation of the MAPK pathway and its enforced expression in renal cancer cells could inhibit *in vitro* migration and invasive traits.

Oncogenic mutations resulting in RAS activation are prevalent in most human tumors, but there are exceptions. RAS mutations in HCC are rare events but paradoxical wild-type RAS activation is common (56). Dietrich et al. (57) discovered that wild-type KRAS expression was increased in HCC compared to non-tumor liver and revealed an inverse correlation with miR-622 expression.

In addition to the above-mentioned examples, several other miRNAs were proven to target and inhibit the expression of RAS oncoproteins (Table 1). These miRNAs are generally downregulated in tumors, thus concurring with reciprocal overexpression and activation of RAS, irrespective of activating gene mutations.

## miRNAs as RAS Effectors

**Figure 1:**
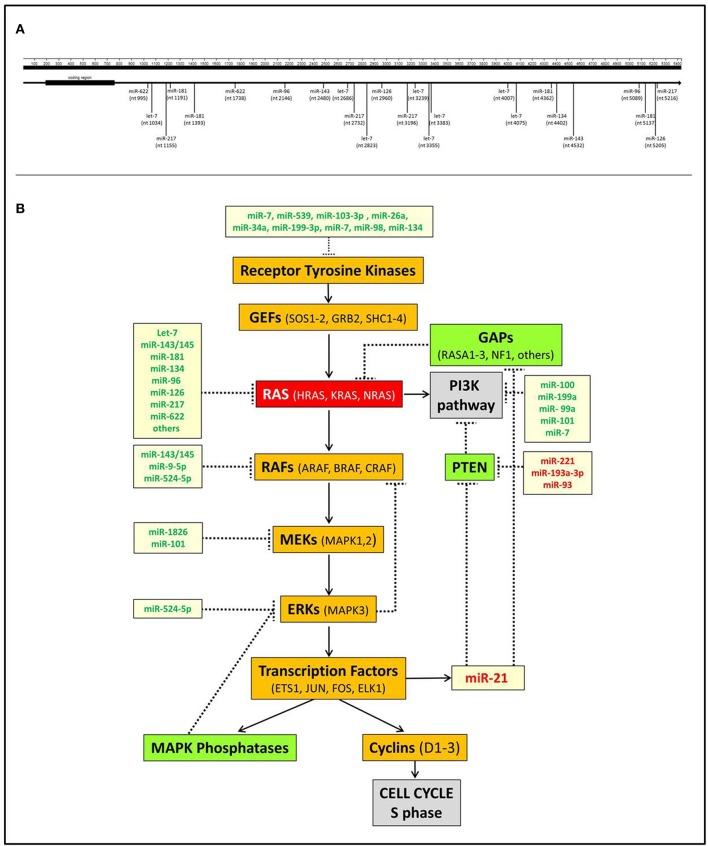
Interactions of miRNAs with RAS. **(A)** Scheme of the direct interactions of miRNAs with the 3′UTR of KRAS. Data were derived from TargetScan v7.2 (http://www.targetscan.org) and from Johnson et al. (18), Chen et al. (40), Jiao et al. (53), Liu et al. (55), and Dietrich et al. (57). **(B)** A simplified scheme of the interplay between miRNAs and RAS pathways. It shows that several miRNAs negatively regulate the MAPK and PI3K RAS-linked pathways at different points. Conversely, miR-21, which is transcriptionally induced by the transcription factor ELK1, inhibits the MAPK and PI3K suppressors GAPs and PTEN, thereby further promoting RAS activation. miRNAs indicated in green are downregulated in tumors, miRNAs indicated in red are upregulated.

The interplay between miRNAs and RAS is not only represented by miRNAs acting as negative modulators of RAS but also includes downstream miRNA effectors. The most significant is undoubtedly miR-21, which is up-regulated by KRAS oncogenic mutants in non-small-cell lung cancer (58), laryngeal squamous cell carcinoma (59), and pancreatic adenocarcinoma (60) as well as many other human cancers. miR-21 is a known oncomiR capable of blocking the expression of tumor suppressor genes antagonists of the PI3K-AKT pathway, such as PTEN, or of the RAS-MAPK pathway, such as PDCD4 or RASA1 (61–63) (Figure 1).

## miRNAs as Regulators of Receptor Tyrosine Kinases (RTKs)

RAS is a crucial node that connects receptor tyrosine kinases (RTKs) with downstream molecular pathways (Figure 1). Hence, miRNAs can affect RAS activity by acting on RTKs as well as MAPK, PI3K, or other pathways.

It is a well-known notion that RAS activation is physiologically triggered by RTKs, a category of transmembrane receptors that become activated in response to growth factors. Several miRNAs are known to target RTK mRNAs and their dysregulation can lead to inappropriate activation of the targeted RTK. Just to mention a few examples, miR-7, miR-539 and miR-103-3p can target and modulate the expression of the epidermal growth factor receptor (EGFR) (64–66); miR-26a was shown to target c-MET, the hepatocyte growth factor receptor (67); miR-199-3p can target the vascular endothelial growth factor receptors 1 and 2 and the VEGFA ligand (68); miR-7 and miR-98 can target the insulin growth factor receptor gene (64, 69).

All the above-mentioned miRNAs were found dysregulated in a variety of human cancers. miR-539 is downregulated in breast cancer (BC) tissues and cell lines. miR-539 enforced expression could inhibit BC cells proliferation and tumor growth *in vitro* and *in vivo* (65). miR-7 is downregulated in breast and colorectal cancer (CRC) cells (64, 66) and its reduced expression in BC patients correlated with higher stage, grade, and poor prognosis (64). The tumor suppressor activity of miR-103-3p was confirmed by the anti-proliferative effects after its enforced expression in lung cancer cell lines; furthermore, the downregulation of miR-103a-3p in NSCLC was associated with poor prognosis (66). miR-26a reduced levels were associated with poor prognosis in Hepatocellular carcinoma (HCC) (67). MiR-26a can also control the expression of VEGFA in HCC cells and impairs VEGFR2-signaling thereby controlling angiogenesis. miR-199-3p, another miRNA that can target VEGFR1, VEGFR2, and the ligand VEGFA (68), is frequently down-regulated in HCC and it has been shown to have *in vitro* and *in vivo* anti-tumor activity in HCC models (68, 70). MiR-98 is down-regulated in retinoblastoma, where it also represents a prognostic marker (69).

The above-reported miRNAs are just a few examples to show how their deregulation can lead to RTKs overexpression and consequently activation of RAS and its downstream pathways. The latter are themselves regulated by miRNAs, whose deregulation may directly cause the activation of RAS downstream effectors independently from RAS triggering.

## miRNAs as Regulators of MAPK Pathway

The MAPK pathway is a well-studied pathway that promotes cell proliferation and is controlled by RAS activation. It includes several effectors with oncogenic function, widely studied in different types of tumors and whose mutations also represent tumorigenic mechanisms.

BRAF is probably the most studied element of the MAPK pathway. BRAF activation has been associated with a missense mutation V600E, commonly found in melanoma and thyroid cancer, but also present at low frequency in several other types of human cancer (71). As expected, various miRNAs can target and regulate BRAF expression. KRAS targeting miR-143 and miR-145, that we have mentioned above, can also target BRAF, indicating a very important role of these miRNAs in regulating the MAPK pathway at several levels (44). As mentioned earlier, these miRNAs are frequently downregulated in various types of cancer. miR-9-5p is another miRNA targeting BRAF, which was shown to be down-regulated in papillary thyroid carcinoma (65).

Further downstream of MAPK pathway cascade, MEK1/MEK2 (also called (MAP2K1 and MAP2K2) as well as ERK1/ERK2, are also targets of miRNAs. miR-1826 can target MEK1. It is down-regulated in bladder cancer and its reduced expression is associated with more severe pathological traits (pT and grade) (72). miR-101 can also target MEK1. This miRNA exhibits reduced expression in diffuse large B cell lymphoma (DLBCL) and it is associated with a worse prognosis (73). miR-665 has been also shown to indirectly activate MEK in BC cells by targeting the nuclear receptor subfamily 4 group A member 3 (NR4A3) gene. This miRNA is upregulated in breast cancer where its upregulation is associated with metastasis and poor survival (74).

## miRNAs That Act on Multiple Targets of the RAS Pathway

Among the several miRNAs that regulate elements of the RAS-centered pathways, some miRNAs target multiple genes belonging to the pathway thus reinforcing their role in modulating MAPK pathway activation.

In this respect, miR-134 is a typical example, as its target genes not only include KRAS (75), but also EGFR (76), HER2 (77), STAT5B (54), and PIK3CA (78), which are upstream and downstream elements of the RAS-centered pathways. This miRNA is downregulated in numerous types of human cancers, where it affects cell proliferation, survival, invasiveness, metastasis, and apoptosis [reviewed in (79)]. This miRNA exemplifies the deregulatory action of single miRNA and consequent wide effects on tumorigenic signals by acting on multiple elements of the RAS pathways (79). Other miRNAs targeting multiple RAS effectors include miR-143 / miR-145, previously mentioned to target all RAS genes and BRAF; miR-524-5p that can target both BRAF and ERK2. In melanoma, miR-524-5p is downregulated and affects cell migration and proliferation both *in vitro* and *in vivo* (80).

These miRNAs are potentially very important, as they can represent useful molecules to effectively restore the normal expression of multiple proteins belonging to RAS pathways.

## microRNAs Implicated in Resistance to Target Therapies

Therapeutic interventions in advanced cancers include traditional chemotherapy as well as targeted/immuno-therapies. Targeted therapies make use of molecules capable of blocking aberrantly activated oncogenes that act as tumorigenic drivers. Oncogenic RAS proteins would represent outstanding targets for such therapies. But, no drug targeting RAS has been yet validated for clinical use. At present, most available targeted therapies are instead designed to block the activity of several elements of RAS-centered pathways. These include a large number of tyrosine kinase inhibitors (TKIs) or antibodies against RTKs; drugs that target BRAF V600E mutation (vemurafenib and dabrafenib), MEK (trametinib, cobimetinib and binimetinib), PI3K mutations (alpelisib), and mTOR (everolimus). The RAS pathways are therefore targeted by several drugs, with the RAS itself being a major exception.

Even more disappointing is the fact that mutant activated RAS often reduces the efficacy of targeted drugs and patients become resistant to therapies. One of the best-known mechanisms associated with the emergence of TKI resistance is indeed KRAS mutation. It is known that tumors with KRAS mutations at codons 12, 13, 61, or 146 do not respond to treatment with anti- EGFR antibodies or TKIs and therefore mutational analyses on all RAS genes are carried out on tumor biopsies before a therapeutic regimen is chosen.

Albeit not implemented for clinical use, given their important role in regulating RAS and linked pathways, it is reasonable to believe that altered miRNA expression could also affect the development of resistance to targeted therapies. To this effect, a number of experimental evidences exist (81–95).

Among miRNAs that target KRAS, the reduced expression of miR-181a was shown to be associated with gefitinib resistance in lung cancer (96, 97); in CRC patients treated with cetuximab, it was reported that low levels of miR-181a were associated with a lower overall survival, indicating a reduced efficacy of anti-EGFR therapy (98). While miR-145 was shown to synergize with cetuximab activity (99), high levels of let-7 could predict the efficacy of cetuximab therapy even in CRC patients carrying mutant KRAS (100).

Dietrich et al. (57) not only revealed an inverse correlation of KRAS and miR-622 expression but, additionally, they could attribute KRAS-miR-622 interplay to therapy resistance since sorafenib induced further KRAS augmentation and down-regulation of miR-622. These few examples suggest that the miRNA-mediated modulation of RAS protein levels can indeed affect the response to TKIs or anti-EGFR targeted therapies.

In addition to RAS, the dysregulation of miRNAs responsible for the activation of elements of the MAPK or the PI3K pathways can also reduce the efficacy of TKIs. For example, the reduction of PTEN protein level by up-regulated miRNAs, like miR-21, miR-221, miR-23a and miR-214, can reduce efficacy of TKIs in lung cancer by activating the PI3K pathway (83, 101–106). miRNAs have also been associated with resistance to the BRAF inhibitor vemurafenib (107–110). In short, several studies have shown that the dysregulation of miRNAs has an important role in the efficacy of target therapies, thus suggesting that their levels of expression can be useful to guide the choice of therapy, alongside the more conventional mutational investigations. Furthermore, they also provide suggestions for potential therapeutic approaches useful to restore or improve sensitivity to treatments.

## Conclusions

Taken together, published data provides a strong indication that altered miRNA expression represents an important mechanism for RAS activation, with various implications. First, it represents a mechanism of pathogenic relevance, responsible for the promotion of several tumor traits, irrespective of RAS oncogenic mutations. Second, considering that the activation of RAS represents a frequent mechanism of resistance for drugs directed against RTKs, it is possible that miRNA dysregulation represents a relevant aspect to consider when assessing the proper management of patients on target therapies. Third, miRNAs may represent potentially useful molecules for the control of RAS oncogenic activation, aimed at overcoming the lack of drugs targeting RAS and possibly improving the efficacy of target therapies.

## Author Contributions

RR, LL, RS, and MN contributed to the writing and editing of the manuscript.

### Conflict of Interest

The authors declare that the research was conducted in the absence of any commercial or financial relationships that could be construed as a potential conflict of interest.
